# ATP-Nlrp3 Inflammasome-Complement Cascade Axis in Sterile Brain Inflammation in Psychiatric Patients and its Impact on Stem Cell Trafficking

**DOI:** 10.1007/s12015-019-09888-1

**Published:** 2019-04-24

**Authors:** Mariusz Z. Ratajczak, Aaron Mack, Kamila Bujko, Alison Domingues, Daniel Pedziwiatr, Magda Kucia, Janina Ratajczak, Henning Ulrich, Jolanta Kucharska-Mazur, Jerzy Samochowiec

**Affiliations:** 1 Stem Cell Institute at James Graham Brown Cancer Center, University of Louisville, University of Louisville, 500 S. Floyd Street, Rm. 107, Louisville, KY 40202 USA; 2 Department of Regenerative Medicine Warsaw Medical University, Warsaw, Poland; 3 Department of Biochemistry, Institute of Chemistry, University of São Paulo, Louisville, Brazil; 4 Department of Psychiatry, Pomeranian Medical University, Szczecin, Poland

**Keywords:** Sterile inflammation, Purinergic signaling, Nlrp3 inflammasome, Complement cascade, Psychiatric disorders, Stem cell mobilization

## Abstract

Recent evidence indicates that the occurrence of psychiatric disorders in patients is linked to a local “sterile” inflammation of brain or due to a systemic inflammation process that affects the central nervous system. This is supported by the observation that in peripheral blood of psychotic patients are detectable several mediators and markers of inflammation as well as clinical data on correlations between systemic chronic inflammatory processes and psychiatric disorders. This may explain why some reported anti-inflammatory treatment strategies have beneficial effects on ameliorating psychotic events. In this review we will present a concept that aberrant purinergic signaling and increases in extracellular level of adenosine triphosphate (ATP) in the brain parenchyma may lead to activation of Nlrp3 inflammasome in microglia cells and as a consequence microglia released danger associated molecular pattern (DAMP) proteins activate complement cascade (ComC) in mannan binding lectin (MBL) – dependent manner. Activation of ATP-Nlrp3 inflammasome-ComC axis may also orchestrate trafficking of stem cells released from bone marrow into peripheral blood observed in psychotic patients. Based on this, the ATP-Nlrp3 inflammasome-ComC axis may become a target for new therapeutic approaches, which justifies the development and clinical application of efficient anti-inflammatory treatment strategies targeting this axis in psychiatry.

## Introduction

Mental or psychiatric disorders remain a significant global health problem. According to World Health Organization these pathologies could be divided based on their clinical classification and worldwide frequency into i) depression (~300 million cases), ii) bipolar disorder (~60 million cases), iii) dementia (~50 million cases), and iv) schizophrenia and other psychoses (~23 million cases) (https://www.who.int/en/news-room/fact-sheets/detail/mental-disorders). The etiology of mental disorders is still not completely understood, although it has been speculated to be a combination of environmental, stress and genetic factors. From clinical point of view, mental diseases may occur as a single episode in a patient, or be persistent, relapsing and remitting.

Since the exact causes of mental disorders are often unclear, several theories have been proposed including usage of i) some brain damaging substances and drugs including alcohol, caffeine, amphetamines and cannabis, ii) changes in personality of affected individuals such as emotional instability or pessimism supported by family history of such behavior, as well as iii) environmental and social factors including social stress, discrimination and childhood trauma [1–4]. In further detail below, we will discuss all these brain affecting noxiae and stress stimuli which may lead to “sterile” inflammation of the brain.

Therefore, this review will focus on available literature and our own data allowing for an emerging picture of the interplay between the mental stress- or inflammation-mediated activation of innate immunity, as a mechanistic basis for the occurrence of psychiatric disorders. Based on this we will provide supporting evidence that the occurrence of psychiatric disorders is linked to the presence of sterile inflammation induced primarily in the brain tissue or as result of systemic inflammation process in other organs that may affect the central nervous system [5–7]. It is well known that patients suffering from mental disorders often exhibit inflammation-related abnormalities in the peripheral blood, including elevated levels of circulating pro-inflammatory cytokines and chemokines, increased numbers of circulating monocytes and neutrophils, as well as enhanced reactivity of microglia, astrocytes, and brain endothelial cells to various pro-inflammatory signals [5–14].

Therefore, the better understanding of abnormalities in purinergic signaling within the brain, identification of links between purinergic mediators and Nlrp3 inflammasome in microglia cells and activation of complement cascade (ComC) by products released from cells in Nlrp3 inflammasome-dependent manner provides a basis for identification of ATP-Nlrp3 inflammasome-ComC axis as potential trigger mechanism involved in pathogenesis of some mental diseases [15]. Activation of ATP-Nlrp3-inflammasome-ComC axis may be also responsible for egress of stem cells from bone marrow into peripheral blood observed in psychotic patients [16].

Identification of this pro-inflammatory axis justifies new treatment strategies in psychiatry that are based on the application of anti-inflammatory drugs [17]. We will present current data as well as speculate on other potential anti-inflammatory treatment strategies that could be developed and employed in the clinic.

## Development of Innate and Adaptive Immune and their Potential Link to Central Nervous System

In humans, the immune system operates based on the presence of innate and adaptive immunity [18]. The innate immune system arose during evolution ~600 million years ago and operates in primitive multicellular organism, fungi, plants and vertebrates including humans [18]. This immune system consists of a cellular and humoral arm. The cellular arm involves several type of cells including neutrophils, monocytes, basophils, eosinophils, mast cells, natural killer cells and dendritic cells that secrete several soluble mediators. Some of these cells also possess phagocytic activity and originate mainly in the bone marrow and circulate in peripheral blood and are patrolling peripheral tissues to detect potential invading organisms or damaged cells. As we will discuss bellow, one can consider brain microglia as a part of extended innate immunity cellular arm that extends into central nervous system [14, 19].

The humoral arm of innate immunity consists of proteins that may activate ComC in i) classical-, ii) mannan binding lectin-, and iii) alternative-pathway [18]. It is also characterized by circulating in peripheral blood molecular pattern recognition receptors (PRR) such as collectins, ficollins and pentraxins [5, 18]. The main task of innate immunity is immune surveillance by the cellular and humoral arm to detect specific molecular patterns present in invading microorganisms or in the host organism’s own damaged tissues that are not present in healthy tissues. Therefore, the innate immune system mediates inflammation as a physiological response to i) insults, ii) infections, and iii) biological stressors [5, 18, 20]. Its major tasks is i) recruitment of immune cells to sites of infection and tissue damage, ii) activation of the ComC to remove bacteria, activate cells, and promote clearance of antibody complexes or dead cells as well as iii) identification and removal of foreign substances by specialized white blood cells endowed with phagocytic properties [18].

By contrast to innate immune system, adaptive immunity system based on specialized T lymphocytes and antibody producing B lymphocytes appeared later, ~500 million years ago, and unlike innate immunity depends on adaptive responses against antigens that have been first recognized and subsequently presented to adaptive immunity cells by the cells belonging to innate immunity [18, 20]. Activation of the adaptive immune system occurs through a process known as antigen presentation.

In comparison to innate and adaptive immunity, the central nervous system arose ~550 million years ago, and thus both nervous and immune systems have co-evolved during evolution and are in constant crosstalk and communication. To support this co-evolution, brain microglia cells that are involved in clearance of pathogens and damaged neural cells from the brain show their evolutionary development as an unique interaction between the central nervous system and innate immunity [14, 19, 21]. Furthermore, as recently acknowledged, the ComC pathway plays an important role in developing the brain in the process of synaptic pruning to remove non-efficient or excessive synaptic connections [14, 19]. Finally, what is relevant for the topic of this review are some mediators such as ComC proteins belonging to humoral arm of innate immunity may be also synthetized locally in the brain [22–24]. This local synthesis is important because blood-brain barrier prevents penetration of ComC macromolecules synthesized in liver into brain parenchyma. Therefore, local production of ComC proteins by cells resident in the brain is crucial to maintain the local innate immunity defense system [23]. It is well known that both microglia cells and astrocytes synthesize ComC proteins involved in activation of classical and alternative pathway of ComC as well as proteins involved in terminal activation of ComC along with ComC regulating proteins [23]. What is important for this review it has been shown that leptomeningeal cells secrete mannan binding lectin (MBL) involved in activation of MBL-dependent pathway of ComC [24]. All this data supports a developmental and functional link between innate immunity and central nervous system and supports a role innate immunity plays in pathogenesis of psychiatric disorders.

## Aberrant Purinergic Signaling as Potential Initiator of “Sterile” Brain Inflammation in Psychiatric Disorders

**Fig. 1:**
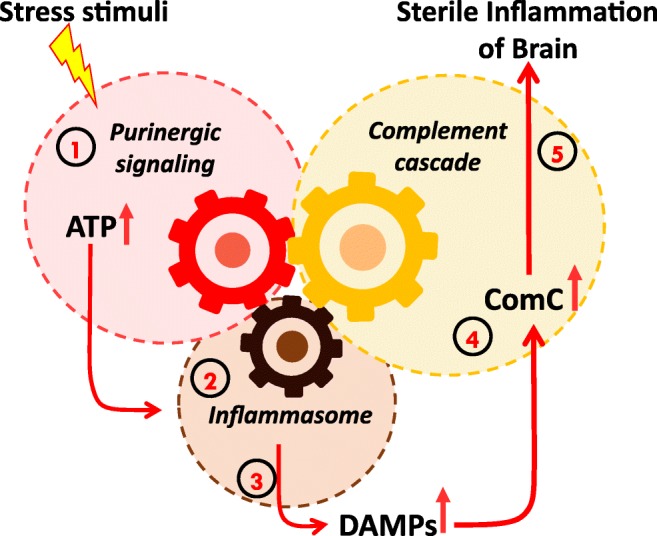
**The inflammasome as a gear or cogwheel that couples purinergic signaling with the complement cascade (ComC) in sterile inflammation of the brain**. Increase of extracellular ATP in the brain tissue in response to stressors (1) activates via P2X7 receptor microglia that respond by activation of Nlrp3 inflammasome (2). As result of inflammasome activation several DAMPs are released, including Hmgb-1 and S100a9 (3), which are recognized by circulating mannan binding lectin (MBL) (4) and activate the ComC in the MBL-dependent pathway. Activation of the ComC leads to release of C5 cleavage fragments that are crucial to maintain inflammation state in the brain parenchyma (5)

Sterile inflammation in contrast to inflammation caused by pathogens, is triggered by physical, chemical or metabolic stimuli and the central nervous system is an anatomical place where it can occur [5]. Therefore, the main concept presented in this review is that excessive release into intercellular space of adenosine triphosphate (ATP) from the activated/damaged neurons or aberrant purinergic signaling, triggers activation of Nlrp3 inflammasome in microglia and astrocytes [25, 26]. As a consequence, these cells secrete some danger associated molecular pattern molecules (DAMPs) that activate ComC. This sequel of events induces sterile inflammation in the brain parenchyma (Fig. 1) and as postulated in this review, the ATP-Nlrp3 inflammasome-ComC axis emerges as a culprit in some mental disorders.

Purinergic signaling is one of the neurotransmitter systems involved in signaling in central nervous system [25]. Mediators of purinergic signaling employ an evolutionarily ancient signaling mechanism that regulates beside neurotransmission several aspects of brain cell biology, such as neurodevelopment, neurodegenerations and repair and provide signals for chemotaxis and modulation of the responsiveness of innate and acquired immune cells to inflammatory cues. Extracellular ATP is its major mediator and is released in increased concentrations from activated, stressed and dying cells [26]. Beside ATP, the others involved in purinergic signaling are its metabolites, such as ADP, AMP, and the nucleoside adenosine.

Purinergic signaling mediators interact on the surface of cells with P2 nucleotide- and P1 nucleoside-activated receptors [25]. P2 receptors are further subdivided into metabotropic P2Y and ionotropic channel P2X receptor families based on structural characteristics. The P2Y receptor family includes eight G protein-coupled receptors (P2Y1, 2, 4, 6, 11, 12, 13, and 14) activated mainly by ATP but also but depending on the involved P2Y receptor subtype by some other mediators including ADP, UTP, UDP or UDP-glucose. The P2X ionotropic channel receptor family consists of trimeric homomeric or heteromeric receptors formed by seven subunits (P2X1, 2, 3, 4, 5, 6, and 7), which are activated by ATP [25]. In contrast the P1 receptor family consists of four G protein-coupled receptor subtypes, A1, A2A, A2B, and A3, which are activated by ATP metabolite – adenosine [25]. Purinergic signaling is regulated by diversity ATP-hydrolyzing enzymes expressed on cell surface as receptors (CD39 and CD73) [25]. What is interesting lithium that is employed in treatment of bipolar disease affects also extracellular nucleotide degradation [27, 28].

This large number of purinergic receptors and ligands as well as enzymatic cascades for converting extracellular ATP into adenosine shows the complexity of this signaling system. A recent excellent review in a comprehensive way presents involvement of aberrant purinergic signaling in pathogenesis of major depressive disorder, schizophrenia, bipolar disorder, autism, anxiety and attention deficit/hyperactivity disorders [27]. All these pathologies could be tracked back to defects in signaling from P1 and P2 receptors as well as enzymes processing metabolism of purinergic mediators [27].

**Fig. 2:**
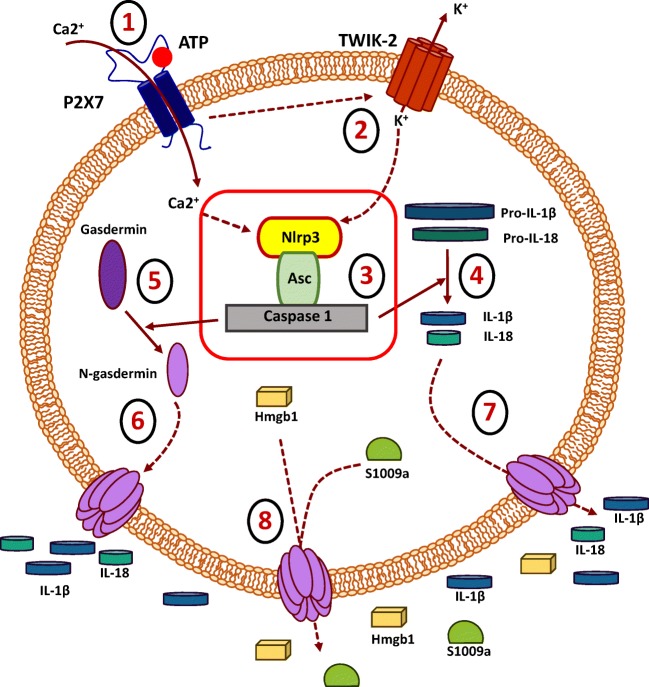
**The most important steps in the intracellular activation of NRLP3 inflammasome by extracellular ATP**. Extracellular ATP activates P2X7 on microglia cells (step 1) which subsequently activates K+ efflux channel TWIK-2 (step 2). A decrease in K+ intracellular levels triggers the activation of the NRLP3 inflammasome complex (step 3). In response to this caspase, 1 cleaves pro-IL-1β and IL-18 to active ready for secretion IL-1β and IL-18 (step 4), and in addition cleaves gasdermin that releases N-gasdermin (Step 5) that insert into the cell membrane to create pores (step 6) for the release of IL-1β and IL-18 (step 7) as well as DAMPs (step 8)

Among family of P2X inotropic receptors, the P2X7 receptor seems to be the most important in inducing sterile inflammation of brain. P2X7 receptor similarly as other receptors from P2X family is activated by three ATP molecules that bind to each of his three subunits and open the ion-permeable channel pore influx into cells of Na^+^ and Ca2^+^ cations [29]. What is important for topic of this review ATP-P2X7 interaction and subsequent influx of Ca2+ trigger activation of Nlrp3 inflammasome (Fig. 2). Because activation of Nlrp3 inflammasome requires efflux of K^+^ from the target cells, ATP stimulated P2X7 activates two-pore domain K^+^ efflux channel TWIK2 that by promoting release of potassium from the cells triggers inflammasome activation [29].

## Microglia Cells and their Role in Initiating Sterile Inflammation of the Brain

Microglia are cells that sense and respond to stress related noxiae and are considered to be resident macrophage population of the central nervous system [14, 19]. They express several receptors including those involved in purinergic-, chemokine-, cytokine- and ComC cleavage products- (e.g., C3a and C5a) signaling [14, 19, 21].

Overall, microglia belong to glial system of non-neuronal cells and their relationship to cellular arm of innate immunity has been discussed earlier in this review. They account for 5–20% of all glia cell population in central nervous system parenchyma and play an important role in brain development by regulating proliferation and differentiation of neural cells as well as are involved in the formation of synaptic connection by phagocytic removal of redundant or less effective in signaling synapses [14, 19]. This process is called synaptic pruning and occurs during brain maturation. Nevertheless, it may occur later on in adult life in some pathologic conditions (e.g., neurodegeneration or schizophrenia). Microglia can also phagocyte in brain damaged cells playing important role in maintaining integrity and repair of this organ [14, 19].

The developmental origin of these cells in the central nervous system has been disputed in the past [21]. It has been speculated that they are bone marrow-derived and in steady state conditions originating from monocytes, migrating with the blood stream and settle down in the brain. Taking this concept into consideration they were supposed to be able to cross easily blood-brain barrier. Recently, it has been proposed that microglia precursors originate at stage of primitive hematopoiesis in yolk sac blood islands and colonize at early stages of embryogenesis developing brain [14, 19]. Therefore, it is likely that microglia in adult life in steady state conditions are supplied by local progenitors residing in brain parenchyma. Nevertheless, during brain inflammation or injury microglia could be still derived from bone marrow-derived monocytes that migrate into brain tissue across weakened and permeabilized blood-brain barrier [14, 19].

What is very well known is microglia in response to ATP and other pro-inflammatory stimuli such as C3a, C5a ComC cleavage fragments, several cytokines and chemokines may activate intracellular Nlrp3 inflammasome complex [16]. The involvement of ATP-P2X7 axis in activating Nlrp3 inflammasome in microglia by promoting K^+^ efflux by TWIK2 receptor is schematically depicted in Fig. 2. Moreover, since P2X7 receptor is also highly expressed by astrocytes it may activate in these cells Nlrp3 inflammasome as well [27]. Recently however, it has been demonstrated that ATP response of microglia cells is regulated in addition to P2X7 receptor, by another P2Y12 purinergic receptor that activates two pore domain K^+^ channel THIK-1 [29]. Therefore, still more work has to be done to address if other ATP stimulated purinergic receptors (e.g., as postulated P2Y2 and P2Y14) could be involved in activation of Nlrp3 inflammasome in microglia cells and astrocytes [27].

## Nlrp3 Inflammasome as a Cogwheel/Gear that Connects Purinergic Signaling with ComC in Inducing and Maintaining “Sterile” Inflammation of the Brain

As it is shown in Fig. 1 extracellular ATP stimulated P2X7 receptor and activates Nlrp3 inflammasome which serves as a gear or cogwheel to connect purinergic signaling with ComC to induce sterile inflammation in brain tissue. To support this notion activation of Nlrp3 inflammasome has been already demonstrated to be involved in major mental disorders [15].

**Fig. 3:**
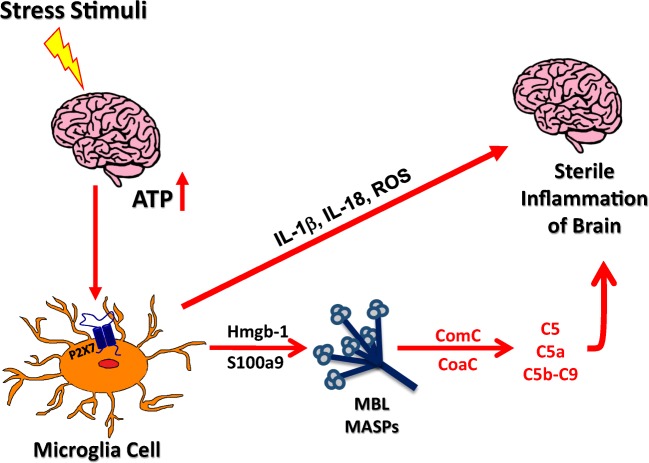
**The interplay between purinergic signaling and ComC activation during sterile brain inflammation.** Increase in extracellular ATP secreted level in brain tissue stimulates via P2X7 receptor Nlrp3 inflammasome in microglia cells. Activated microglia secrete IL-1, IL-18 and ROS that promote sterile inflammation in brain parenchyma. Microglia cells also release HMGB1 and S100a9 that as DAMPs activate MBL pathway of ComC activation. Release of ComC proteins cleavage fragments such as C3a and C5a anaphylatoxins maintains sterile inflammation state of brain

Nlrp3 belongs to the inflammasome family that consist of several members which also includes Nlrp1, NlrC4 and AIM2 inflammasomes [30]. Overall, inflammasomes are multiprotein oligomer complexes that are important components of the innate immunity network. They may be triggered by danger-associated molecular pattern molecules (DAMPs) also known as alarmins, which are released by activated or damaged cells [5, 31, 32]. The most important DAMP that activates Nlrp3 inflammasome via P2X7 receptor is extracellular ATP (Fig. 2). Activation of Nlrp3 inflammasome leads also to secretion of other DAMPs such as high molecular group box 1 (Hmgb1) and S100a9 proteins. These proteins are important because as shown in Fig. 3 they bind to circulating collectin MBL and activate ComC in MBL-dependent manner.

What is also important to mention Nlrp3 inflammasome in microglia cells could be also activated by reactive oxygen species (ROS) that are released from mitochondria in response to some stressors [33, 34]. The role of oxidative stress and involvement of ROS has been reported in pathogenesis of anxiety disorders, depression, schizophrenia and bipolar disorder. Interestingly, neuronal P2X7 receptor-induced ROS production has been reported to cause nociceptive behavior in mice [33].

Inflammasomes may also respond and become activated during infections in response to pathogen-associated molecular pattern molecules (PAMPs) [30]. It is important to mention that in addition to inflammasomes that activate inflammation, there are such that have opposite effect (NLRP2, NLRC3, NLRP6, NLRP7, NLRP10, NLRP12, and NLRX1) [16, 30]. The potential role of these inflammasomes in sterile inflammation of the brain (protective?) requires further studies. In meantime, the NLRP3 inflammasome currently the best-characterized member is expressed in cells belonging to cellular arm of innate immunity including i) granulocytes and monocytes, ii) dendritic cells and iii) microglia. It is composed of several proteins, including NLRP3, CARD-containing adaptor (ASC), and caspase 1, and is responsible for the activation of inflammatory responses in innate immunity cells including microglia as shown in Fig. 2 [16]. ATP-P2X7 interaction activates intracellular enzyme caspase 1 that promotes by proteolytic cleavage of pro-IL-1β and pro-IL18 maturation and secretion from cells biologically active interleukin 1β (IL-1β) and interleukin 18 (IL-18) [30]. Mature form of both cytokines are released into the extracellular space and this process is facilitated by the creation after Nlrp3 inflammasome activation of cell membrane pores due to insertion into the membrane another caspase-1 cleavage product, N-gasdermin, that is, gasdermin protein fragment (Fig. 2). In fact elevated level of IL-1β and IL-18 in biological fluids is a sign on Nlrp3 inflammasome activation and has been reported as useful diagnostic marker in several mental disorders [16].

IL-1β and IL-18 that are secreted from microglia cells may by employing autocrine positive feedback loop maintain their prolonged auto-activation. Therefore, an important question is if there are some mechanisms that control over-activation of microglia cells. To address this question there are two recently described intrinsic enzymatic pathways including heme oxygenase-1 (HO-1) and inducible nitric oxide synthetize (iNOS) that are inhibitors of Nlrp3 inflammasome and they may control its over-activation [35, 36]. To support this, HO-1 activators which possess anti-inflammatory effects have been proposed to treat psychiatric patients [37, 38].

Beside interleukins, there are secreted from microglia DAMPs such as Hmgb1 and S100a9 that provide an important link to activation of ComC (Figs. 1 and 3). As mentioned both DAMPs are recognized by circulating in biological fluids including cerebrospinal fluid soluble PRR - collectin MBL [24]. Binding of MBL to DAMPs activates mannan activated serum proteases (MASPs) that activate ComC in MBL-dependent pathway [18]. Similarly as IL-1β and IL-18, ComC products including anaphylatoxins C3a and C5a can again propagate sterile inflammation in brain by activating specific receptors (C3aR and C5aR) on surface of microglia cells. To control this process activation of ComC is controlled by several ComC inhibitors that are expressed in brain tissue by microglia and astrocytes. Moreover, activation of ComC is also negatively regulated by mentioned above HO-1 and iNOS [35, 36].

## Implications of ComC and Collateral Coagulation Cascade Activation in Brain Parenchyma

ComC belongs as mentioned above to humoral arm of innate immunity and over 30 proteins and protein fragments including serum proteins and cell membrane receptors make up ComC system [18]. These proteins account for about 10% of the globulin fraction of blood serum and concentration of the most abundant ComC protein - C3 is 1 mg/1 ml in blood plasma. As mentioned above several ComC proteins are synthesized in CNS and several receptors that respond to these proteins as well as inhibitors of ComC activation are expressed in brain parenchyma [23]. Therefore, ComC plays an important role in neurodevelopment and in brain maturation by removing (pruning) excessive or signaling non-effective synapses [14, 19].

We postulated as it is shown in Fig. 3 that ComC cleavage fragments contribute to sterile inflammation of the brain and thus play an important role in psychotic disorders [8–10, 37, 38]. To support this notion, our group demonstrated increased concentrations of C3a and C5a anaphylatoxins in peripheral blood in 30 patients suffering from bipolar disorder for at least 10 years, who were not treated with lithium salts as compared to healthy age and sex matched controls [10]. This suggests involvement of the ComC in the pathogenesis of bipolar disorder, and provides further evidence for immune system dysregulation in these patients. As proposed, C5a may activate microglia, astrocytes, and endothelial cells in brain tissue.

However, ComC becomes activated in brain tissue in MBL-dependent pathway, its activation could be further amplified by alternative pathway of ComC activation. The role of MBL in this process is supported by observations that i) MBL is synthesized in the brain by leptomeninges, ii) it is present at high concentration in cerebrospinal fluid, and iii) activates ComC in several cerebral pathologies [22, 24]. As it is shown in Fig. 3, MBL binds DAMPs (Hmgb1 and S100a9) secreted by microglia, astrocytes and damaged neurons. Once bound to these ligands, MBL recruits MBL-associated serine proteases (MASP-1 and -2) [32]. In the next step MASP-1 initiates activation of the ComC. MBL-recruited MASPs cleave C3 and C3 cleavage products and initiate the generation of classical C5 convertase, which subsequently cleaves C5 into the anaphylatoxin C5a and iC5b.

In parallel, MASP-1 also activates prothrombin, giving rise to thrombin, which has, as reported, C5 convertase-like activity [39]. This release of active thrombin due to MBL-MASP pathway of ComC activation may explain why in psychotic patients very often is observed activation of coagulation. It has been reported that mental stress affects coagulation, and severe mental illnesses, such as recurrent depression and schizophrenia, are associated with an increased thrombosis risk and cardiovascular morbidity [11].

What is important the end products of ComC activation, the anaphylatoxins C5a and desArgC5a as well as C5b9 (also known as the membrane attack complex, MAC) perpetuate inflammation in the surrounding neural tissues by activating cells through interaction with specific surface receptors present e.g., on microglia cells and astrocytes (C5a, desArgC5a) or damage cell integrity in the brain (MAC) [18, 21–23]. This process occurring in brain as part of prolonged sterile inflammation may lead to psychotic disorders. Furthermore, in those all cases in which the brain is exposed to activated ComC mediators circulating in blood due to a systemic inflammatory disorder, these mediators may potentially penetrate the blood–brain barrier, in particular when this barrier is damaged and permeabilized, and thereby directly affect neural tissue.

## Psychoses and Increase in Circulating in PB Stem Cells

**Fig. 4:**
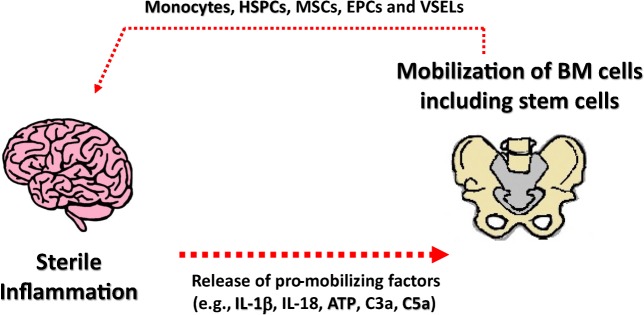
**Crosstalk between sterile inflammation of brain and bone marrow.** Several pro-mobilizing factors released form brain during sterile inflammation stimulate bone marrow to release monocytes, HSPCs, MSCs, EPCs and VSELs. Some of these cells may enter brain parenchyma due to damaged and leaky brain-blood barrier

It has been reported that the numbers of circulating leucocytes and monocytes increase in peripheral blood during exacerbation of psychotic symptoms [8–10]. This supports the presence of inflammation and helps to explain this phenomenon since both IL-1β and IL-18 and ComC cleavage fragments are known to be able to increase the number of circulating leucocytes and stem cells in the blood (Fig. 4). Our team found that in addition to observed leukocytosis, there are also changes in the number of circulating stem cells [8–10]. We found this to be true for several types of stem cells, such as hematopoietic stem cells (HSCs), multipotent stromal cells (MSCs), endothelial progenitor cells (EPCs), and some rare very small embryonic-like stem cells (VSELs). It is well known that under steady-state conditions there are always some stem cells detectable at very low levels circulating in peripheral blood, including mainly HSCs but also MSCs, EPCs, and very rare VSELs [8–10]. The numbers of these stem cells increases in circulation during stress and pathological situations that are related to inflammation and tissue damage [40–44] and this explains their increase in circulating blood in psychotic patients.

We found that the pattern of cells released into the circulation as well as the profile of chemoattractants detected in peripheral blood differs between various disorders and may be potentially helpful as a diagnostic or a prognostic tool [8–10]. However, we are aware that further studies are needed to understand the clinical implications of this phenomenon.

To explain a meaning of circulating stem cells in psychotic patients there are some indications that certain processes occur during psychotic disorders that are related to brain tissue remodeling as seen for example in depression, and stem cells could play a role here [8–10]. Circulating stem cells may also be a source of paracrine soluble factors (cytokines, growth factors, chemokines and bioactive lipids) as well as extracellular microvesicles that may deliver their content (e.g., mRNA, miRNA, proteins, mitochondria) to the brain cells [45]. Moreover, the blood-brain barrier that is tight in steady state conditions could become permeable to circulating stem cells during sterile inflammation in the brain. In future studies it would also be interesting to see how pharmacological as well as other types of treatment in psychotic patients, such as electro- or insulin-shock therapies, affect the egress of these cells from bone marrow into peripheral blood and potentially enforce their trafficking between bone marrow and brain tissue.

In addition to circulating stem cells, we noticed changes in important factors that beside IL-1β and IL-18 direct their trafficking, including ComC cleavage fragments and bioactive phospo-sphingolipids, which may be employed as a new diagnostic and prognostic tools in psychiatry.

## New Treatment Strategies of Sterile Inflammation of the Brain

Based on clinical and molecular evidence that sterile inflammation of the brain leads to psychotic disorders anti-inflammatory strategies have already been employed in clinical settings [17, 46]. The most important anti-inflammatory administered thus far to patients include Minocycline, Celecoxib, Dicloflenac and COX-2 inhibitors [17]. These anti-inflammatory drugs have been used alone or in combination with selective serotonin reuptake inhibitors (SSRI) e.g., Escitalopram or tricyclic anti-depressants that increase activity of serotonin in brain - e.g., Nortiptiline. The results from first clinical data in patients cohort studies are quite promising [17]. Therefore, an adjunctive therapy with anti-inflammatory medication, especially in cases where conventional therapy has failed to bring complete recovery, may provide a novel therapeutic strategy for psychiatric disorders.

What is important for this review, the anti-inflammatory therapeutic strategies that target ATP-Nlrp3 inflammasome-ComC axis could employ modulators of purinergic signaling (e.g., P2X7 inhibitors), Nlrp3 inhibitors (e.g., MC9950), ComC inhibitors (e.g., HO-1 activators). Some promising data have been reported with some anti-inflammatory adjuvant drug supplements such as curcumin or turmeric [46].

We can expect that accumulating evidence, that supports a role of sterile inflammation of the brain in psychiatric disorders, will lead to development more specific therapeutic anti-inflammatory strategies to inhibit activation of microglia. In these strategies as important emerges ATP-Nlrp3 inflammasome-ComC axis.

## Conclusions

We propose that extracellular ATP-Nlrp3 inflammasome-ComC axis is a driver of sterile inflammation in the brain leading to psychotic disorders. Extracellular ATP activated Nlrp3 inflammasome plays an important role as a gear or cogwheel between purinergic signaling and ComC [16]. The ComC activation is initiated by the MBL–MASP pathway with a supportive role from the alternative pathway of ComC activation. The activation of MBL-MASP pathway of ComC activation is triggered by an increase in DAMP mediators released during inflammation, including extracellular Hmgb1 and S100a9, which are recognized by circulating collectin MBL [5]. This process is also tightly controlled by the anti-inflammatory and Nlrp3-inhibitory action of HO-1 and iNOS [47, 48]. In parallel, during ComC activation subsets of stem cells from bone marrow are mobilized into peripheral blood that may be involved in certain brain-remodeling processes [16, 49] or exert ani-inflammatory effects [50, 51]. All these observations suggest that modulating innate immune responses related to sterile inflammation will enable the development of the novel innovative approaches for the treatment of psychiatric disorders targeting ATP-Nlrp3 inflammasome-ComC axis.
